# Knowledge, attitudes and practices toward risk of basic nosocomial infection control among pediatric healthcare workers: a cross-sectional study in Nanjing, China

**DOI:** 10.3389/fpubh.2025.1599686

**Published:** 2025-07-23

**Authors:** Ruizhe Yang, Wu Yan, Xu Wang, Wei Li, Shengfan Xue, Tingting Wang, Xiaorong Xiang, Qian Li

**Affiliations:** ^1^Department of Public Health, Children's Hospital of Nanjing Medical University, Nanjing, China; ^2^Clinical Medical Research Center, Children's Hospital of Nanjing Medical University, Nanjing, China; ^3^Department of Infection Management Office, Children's Hospital of Nanjing Medical University, Nanjing, China; ^4^Department of Party and Government Office, Children's Hospital of Nanjing Medical University, Nanjing, China

**Keywords:** infection control measures, pediatric healthcare worker, Delphi method, Chinese population, healthcare-associated infections (HAI)

## Abstract

**Background:**

This study was conducted to systematically evaluate the knowledge, attitudes, and practices (KAP) of pediatric healthcare workers (HCWs) concerning risk assessment in basic nosocomial infection control, with a specific focus on identifying key demographic and professional variables that may influence these outcomes.

**Methods:**

A cross-sectional survey was conducted among 126 pediatric HCWs in Nanjing, China. A structured questionnaire on nosocomial infection control was developed using the Delphi method. Expert consensus was assessed using content validity indices (CVI) and Kendall's W. One-way ANOVA identified significant differences in responses to sub-questions. Factor analysis was based on severity and importance ratings. Linear regression examined associations between demographic variables and risk assessment performance. Statistical significance was defined as *P* < 0.05.

**Results:**

One-way ANOVA demonstrated statistically significant variations in responses to 17 sub-questions (*P* < 0.05), reflecting differing perceptions of severity and importance across departments. Factor analysis extracted two distinct factors related to severity (44.055, 29.767%) and one overarching factor associated with importance (51.505%). However, no statistically significant correlations were observed between age, gender, or professional title and knowledge levels regarding infection control, indicating minimal influence of these demographic characteristics on attitudes toward infection control practices.

**Conclusion:**

These findings provide key insights into current knowledge, attitudes, and practices regarding nosocomial infection control among pediatric HCWs in Nanjing, China. The observed differences between departments highlight the need for tailored interventions. Future research should focus on developing and evaluating such targeted strategies to improve infection control, patient safety, and care quality.

## 1 Background

Nosocomial infections (NIs), also referred to as healthcare-associated infections, represent a major public health concern within medical facilities. These infections generally occur in patients 48–72 h following hospital admission and present considerable challenges to the efficient provision of healthcare services (1). Specifically, NIs are linked to extended hospitalization duration, heightened antimicrobial resistance, exacerbation of pre-exist medical conditions, increased financial strain on patients, and elevated mortality rates in more severe cases (2, 3). Studies suggest that ~15% of hospitalized patients contract NIs, which are significantly associated with increased mortality rates (4). In low-income regions, the prevalence of NIs is estimated to be up to threefold higher compared to that in high-income countries, with infants in these areas facing risk levels that are up to 20 times greater (5). Prevalence rates exhibit considerable variation. In high-income countries, they range from 3.5 to 12%, whereas in middle to low-income countries, the rates span from 5.7 to 19.1%. For instance, a multi-center study conducted across multiple provinces in China reported a weighted prevalence ranging from 1.73 to 5.45% (6, 7).

In addition, the economic impact of NIs is considerable. In China alone, the direct economic burden of hospital-acquired infections is estimated to range between $1.5 billion and $2.3 billion annually (8). This imposes a significant financial burden on both healthcare systems and individual patients. Therefore, effective prevention and management of NIs are essential, particularly in developing countries where healthcare resources are often constrained (8). Reducing the incidence of NIs and improving patient outcomes remain key public health priorities that necessitate targeted, evidence-based interventions (1, 9).

According to Kelman's knowledge, attitude, and practice (KAP) theory, knowledge serves as a foundational element for changing practices, while attitude acts as a catalyst for behavioral transformation (10). Therefore, identifying the key factors that significantly influence KAP is critical, as such insights can inform the development of targeted intervention strategies by healthcare administrators responsible for managing NIs. However, there remains a paucity of systematic research examining the association between KAP and NIs among HCWs, as well as the underlying mechanisms linking these variables within this population (11, 12). Nevertheless, existing studies possess notable deficiencies. Firstly, they merely offered an overview of the current KAP levels without adequately addressing the factors that have an impact on them. Secondly, the majority of published reports on KAP have focused exclusively on hand hygiene practices. To the best of our knowledge, no studies have evaluated KAP or identified its influencing factors among Chinese HCWs regarding basic NIs across pediatric hospitals.

Recent multi-continent pediatric infection-control studies support our findings and highlight the global relevance of the knowledge-to-practice gap described by Kelman's theory. In South Africa, only 41% of clinicians at a tertiary children's hospital achieved adequate KAP scores, consistent with the high-risk perception seen among surgical HCWs (13). A 2024 Ethiopian meta-analysis of pediatric wards reported an average practice score of 46%, further confirming this gap (14). However, regional differences, small single-center samples, and limited multi-variable analysis mean that the causes of poor compliance remain unclear. European data are similarly sparse. In Italy, Parmeggiani et al. (15) found strong hand hygiene knowledge among pediatric emergency staff but low use of eye protection during aerosol-generating procedures. A Scottish review showed that pediatric nurses often misunderstand transmission-based precautions, especially for multi-drug-resistant organisms (16). Importantly, neither study examined how departmental culture or perceived risk severity affect behavior—key factors for designing effective interventions. Evidence from the Americas is even more limited. A Brazilian mixed-methods study found that only 38% of pediatric nurses could identify all components of central-line–associated infection prevention bundles, with workload and lack of feedback cited as major barriers (17).

Basic control of NIs, which includes standard precautions, cleaning, disinfection and sterilization, isolation, and aseptic techniques, forms the foundation of hospital infection prevention (18). It plays a critical role in managing healthcare-associated infections, and healthcare facilities have taken proactive steps to meet related system requirements. However, challenges remain due to the widespread involvement across medical activities, shared responsibilities among HCWs, complex implementation processes, and difficulties in oversight. Common issues include inconsistent protocol adherence, inefficient management, and recurring infections (19, 20).

The present cross-sectional survey, conducted at a major children's hospital in China, addresses existing gaps by identifying how pediatric healthcare workers perceive the relative severity and importance of various infection-control tasks beyond hand hygiene. It also determines which demographic and departmental variables independently predict KAP performance when assessed using validated psychometric instruments. Furthermore, this study examines whether risk-severity factors derived through the Delphi method can provide actionable insights to prioritize training initiatives. By integrating an expert-validated instrument with multivariate modeling, this research not only extends the geographic scope of pediatric KAP studies to East Asia but also introduces a refined severity-importance framework that was previously absent in earlier studies from Africa, Europe, and North America. Although numerous studies have examined healthcare-associated infection's KAP, most have focused primarily on hand-hygiene compliance, involved only single professional groups or small convenience samples, and seldom considered the organizational or risk-severity factors that influence behavior. The aforementioned European, African, and South American pediatric studies relied on univariate statistical methods and were therefore unable to explain how departmental cultures affect adherence to infection control protocols. Our cross-sectional survey addresses these limitations by utilizing a Delphi-validated, multi-domain questionnaire that assesses 40 infection-control procedures—from aseptic preparation to environmental decontamination—and integrates a severity-importance matrix analyzed in relation to demographic and departmental variables through multivariate modeling. These findings offer actionable, department-specific targets for future educational initiatives, benchmarking efforts, and quality improvement programs.

## 2 Methods

### 2.1 Study design and participants

A cross-sectional questionnaire survey was conducted in Nanjing from November 28, 2023, to December 31, 2023. The stratified sampling procedure was implemented as follows: (1) In this study involving HCWs, with the support of the Department of Human Resources, the target population was divided into two strata, clinical departments and medical technology departments, to ensure proportional representation. Following this stratification, systematic random sampling was carried out. Eligible participants were assigned unique identifiers based on the following inclusion criteria: (1) official hospital staff members (interns, nurse assistants, and medical students were excluded due to limitations in their capacity to provide certain types of information); (2) possession of valid professional qualification certificates. Participants were selected using a computerized random number generator. HCWs who were on leave during the survey period, as well as non-clinical personnel, were excluded from participation. A total sample size of 126 was proportionally distributed across the strata to ensure balanced representation. This methodological approach minimized potential sampling bias and enhanced the validity of the findings by incorporating diverse perspectives within the target population. All participating HCWs completed the questionnaire voluntarily, resulting in a 100% response rate, and submitted their responses electronically.

### 2.2 Ethical considerations

Our study received ethical approval from the Institutional Ethics Committee (IEC) of Nanjing Medical University Children's Hospital (Ethics Approval Number: 202501004-1). Given that the level of risk to participants in this study does not exceed minimal thresholds, and in accordance with IEC guidelines, written informed consent is not required for procedures that would normally be conducted outside the context of formal research under comparable conditions. Examples of such procedures include interview-based studies and mail or telephone surveys. Accordingly, we submitted a request for a waiver of the written informed consent requirement. This request was reviewed and approved by the Institutional Ethics Committee of Nanjing Medical University Children's Hospital.

### 2.3 Measurement

Based on departmental classification, 126 HCWs were classified into three major categories: internal medicine, surgery, and healthcare specialties from medical technology departments. Comprehensive demographic data were collected, and participants' knowledge regarding fundamental nosocomial infection control risks was assessed using a structured questionnaire specifically developed for this study. The scores for each item were recorded in a systematic and standardized manner.

The questionnaire utilized in this study was developed based on a previously established basic infection control risk assessment index system, which was constructed using the Delphi method. The instrument encompassed key domains including standard preventive measures, cleaning protocols, disinfection practices, sterilization techniques, isolation procedures, and aseptic operations. Furthermore, it incorporated assessments of the importance of associated sub-questions, the likelihood of infection risk occurrence, the potential severity of such risks, and the capacity to effectively respond to infection-related incidents.

The Importance Evaluation component measures the criticality of each infection control practice, with scores ranging from 1 (low) to 3 (high). This dimension facilitates the prioritization of interventions based on their relevance and significance in infection prevention. The Likelihood of Infection Risk evaluates the probability of infection occurrence in the absence of appropriate preventive measures, with scores ranging from 1 (none) to 5 (large), thereby enabling an understanding of the frequency of risk exposure across departments.

The Severity of Infection Risk component assesses the potential consequences once an infection occurs, using a scale from 1 (none) to 5 (serious). This provides insight into the possible health impacts on patients and the burden on healthcare resources, thus informing the development of targeted response strategies. Finally, the Ability to Respond dimension evaluates a department's readiness and effectiveness in managing infection events after they occur, rated on a scale from 1 (very good) to 5 (nothing).

### 2.4 The Delphi method and expert quality control

The Delphi method was employed in this study to systematically develop a structured questionnaire for expert consultation. A panel of 20 national experts from the medical and healthcare sectors was invited to evaluate infection control risks based on 40 secondary indicators. Following the initial round of consultation, questionnaire items were revised, and those exhibiting substantial inter-expert score variability were reassessed in a second round. Experts were permitted to retain or modify their evaluations as deemed appropriate. The questionnaire also required participants to rate their familiarity with each item and provide justifications for their assessments. Additionally, a dedicated comment section allowed experts to suggest modifications. Finally, two members of the research team independently reviewed all completed questionnaires to verify their completeness and accuracy.

The 20 experts involved in this study were affiliated with 17 hospitals and one disease control center across eight regions, including Jiangsu, Guangdong, Hebei, Shandong, Xinjiang, Guangxi, Beijing, and Shanghai. These professionals specialized in hospital infection management, clinical medicine, nursing, and public health, each having a minimum of 5 years of experience in their respective domains. The participants' ages ranged from 36 to 60 years, with an average age of 48.15 ± 6.04 years. A majority served as heads of infection control departments, while others occupied senior leadership roles such as vice presidents, nursing directors, department heads, head nurses, or directors of disease control. On average, the panel had 20.45 ± 7.91 years of professional experience, and 95% possessed over a decade of relevant work experience.

### 2.5 Data collection procedure

With the support of the hospital's human resources department, potential participants were contacted. After confirming the reliability and validity of the questionnaire, web links to the survey instrument and informed consent documents were distributed via email to eligible participants by the research team. The estimated time required to complete the survey was 15 min. Once participants had completed the questionnaire, responses were submitted electronically, and signed electronic informed consent forms were returned via email. All completed questionnaires were subsequently subjected to a systematic review for data analysis, and those found to be incomplete or improperly completed were excluded from the final data-set.

### 2.6 Statistical analysis

Data analysis was conducted using SPSS 19.0 software. Enumeration data were expressed as composition ratios, while measurement data were presented as mean ± standard deviation. The Delphi method was applied to systematically collect expert opinions on the preliminary nosocomial infection control risk assessment questionnaire. The level of expert engagement was reflected by the effective response rate of the consultation questionnaires. Expert authority was quantified using the expert authority coefficient. The degree of consensus among experts was assessed through the coefficient of variation (CV) and Kendall's coefficient of concordance W. One-way analysis of variance (ANOVA) was employed to identify sub-questions with statistically significant differences, followed by factor analysis based on the severity and importance categories. The Kaiser-Meyer-Olkin (KMO) measure and Bartlett's test of spherical were used to evaluate the appropriateness of conducting factor analysis, with a KMO value ≥0.6 and a significance level ≤ 0.05 for Bartlett's test considered acceptable. Based on principal component analysis, together with the scree plot and interpret ability of factors, two main factors were extracted according to severity. These factors were further rotated using the varimax rotation method to determine the variance contribution rate of each factor. Prior to conducting the regression analysis, we carried out hypothesis tests to assess the linearity, independence, normality, and equal variance of the data, which was ensure the validity and reliability of the subsequent linear regression model. Upon confirming that all assumptions were satisfied, we proceeded with the linear regression analysis with factor scores as dependent variables and general demographic information as independent variables, to explore the relationship between participant characteristics and the fundamental risk assessment of infection control. A *P*-value < 0.05 was considered statistically significant.

## 3 Results

### 3.1 Basic characteristics

A total of 126 healthcare professionals were recruited, including 54 (43.2%) from internal medicine, 42 (33.6%) from surgery, and 29 (23.2%) from other departments. The Delphi method was employed to systematically gather expert opinions on the infection control risk assessment questionnaire. A questionnaire return rate of 99% reflected a high level of expert participation. In terms of gender distribution, 103 participants (81.7%) were female and 23 (18.3%) were male. With regard to age, the mean age of the participants was 39.32 years (standard deviation = 6.68), suggesting that the sample was predominantly composed of young and middle-aged individuals.

In terms of occupational distribution, 35 participants (28.0%) were physicians, and 91 participants (72.0%) were nursing and medical technical staff, comprising 84 nurses and seven medical technicians. Regarding professional titles, 40 participants (32.0%) held junior-level positions, 52 participants (41.6%) held intermediate-level positions, and 33 participants (26.4%) held senior-level positions. This distribution ensured comprehensive representation of perspectives across different professional levels concerning infection control risks.

Participants rated their practical experience at 2.59 (SD = 0.57), indicating a moderate level of work experience. Theoretical knowledge was rated at 2.45 (SD = 0.58), reflecting a solid understanding of relevant concepts. Peer knowledge received a score of 2.15 (SD = 0.58), suggesting limited familiarity with colleagues' roles and responsibilities, which may influence the accuracy of infection risk assessment. Intuitive judgment scored 1.84 (SD = 0.68), demonstrating a tendency to rely more on empirical evidence and theoretical knowledge than on intuition.

The mean score for participants' familiarity with the survey content was 3.49 (standard deviation = 0.83), indicating a relatively high level of familiarity with infection control risk assessment. These results suggest a solid foundation for subsequent evaluation processes (Table 1).

**Table 1:** Basic characteristics.

**Characteristic**	**Total (*N* = 126)**
**Gender**	
Male	23 (18.3)
Female	103 (81.7)
Age	39.32 ± 6.68
**Occupation**	
Physician	35 (28.0)
Nurse + medical technician	90 (72.0)
**Professional title**	
Primary	40 (32.0)
Intermediate	52 (41.6)
Senior	33 (26.4)
**Department**	
Internal medicine	54 (43.2)
Surgical	42 (33.6)
Others	29 (23.2)
Practical experience	2.59 ± 0.57
Theoretical analysis	2.45 ± 0.58
Peer understanding	2.15 ± 0.58
Intuitive judgment	1.84 ± 0.68
Familiarity with the content of the survey	3.49 ± 0.83

### 3.2 Single factor analysis of variance selected 17 sub-questions with differences

Table 2 presents 17 sub-questions assessing the severity of infection risks following exposure to various patient types. Scores are reported as mean ± standard deviation (SD), with *F*-values and corresponding *P*-values provided to evaluate inter-group differences. A *P*-value < 0.05 was considered statistically significant.

**Table 2:** 17 sub-questions with differences were selected by one-way ANOVA.

**Sub-questions**	**Internal medicine (*N* = 54)**	**Surgical (*N* = 42)**	**Others (*N* = 30)**	***F***	***P*-value**
Severity of infection after exposure to a patient with a clear or suspected route of transmission	4.24 ± 0.78	4.71 ± 0.74	4.23 ± 1.01	4.66	0.011
Severity of infection risk after exposure to no communicable infectious diseases	2.94 ± 0.88	3.88 ± 1.09	3.67 ± 1.18	10.866	0.000
Severity of infection risk after exposure to non-infectious diseases	2.57 ± 1.02	3.33 ± 1.24	3.43 ± 1.22	7.585	0.001
Severity of infection risk after exposure to patients with intact skin and without performing any invasive procedures	2.54 ± 1.09	3.40 ± 1.40	2.97 ± 1.63	4.977	0.008
Severity of infection risk after occurrence of medical staff with infectious diseases performing medical activities	3.96 ± 0.92	4.61 ± 0.83	4.41 ± 0.97	6.285	0.003
Severity of infection risk after medical activities of medical staff with broken skin on their hands	3.77 ± 1.12	4.52 ± 0.74	4.07 ± 1.14	6.463	0.002
Severity of infection risk in an area where the patient does not live or where the patient only stays for a short period of time	2.87 ± 0.97	3.80 ± 1.10	3.27 ± 1.68	6.808	0.002
Severity of infection risk after contact with intact mucosa without entering sterile human tissues, organs and bloodstream, and without contact with damaged skin and damaged mucosa	3.53 ± 1.07	4.27 ± 0.95	4.20 ± 1.21	6.761	0.002
Severity of infection risk after use of medical fabrics with potential biological contamination risk by patients	3.42 ± 1.20	4.12 ± 1.08	4.24 ± 1.06	6.881	0.001
Severity of infection risk after various types of non-intravascular puncture, injection and other procedures and treatments	3.82 ± 1.13	4.41 ± 0.89	4.42 ± 0.97	4.826	0.010
Severity of infection risk after the occurrence of sterile liquid configuration	3.69 ± 1.24	4.41 ± 1.07	4.25 ± 1.03	5.038	0.008
Severity of infection risk after occurrence of non-sterile liquid configuration	3.00 ± 1.28	3.78 ± 1.29	3.79 ± 1.28	5.317	0.006
The importance of exposure to people with no communicable infectious diseases	2.13 ± 0.65	2.69 ± 0.56	2.27 ± 0.91	8.035	0.001
The importance of performing catheter insertion, puncture, surgery, and other procedures that require maximum sterile barriers	2.76 ± 0.55	2.95 ± 0.22	2.43 ± 0.90	7.072	0.001
The importance of medical activities for medical staff with broken hand skin	2.62 ± 0.63	2.88 ± 0.33	2.47 ± 0.68	5.138	0.007
The importance of areas of medical activity where no patient resides or where the patient stays only briefly	1.74 ± 0.72	2.21 ± 0.65	2.19 ± 0.79	6.145	0.003
The importance of contact with intact mucosa without entering sterile tissues, organs and blood flow of the human body, and without touching items with damaged skin and damaged mucosa	2.38 ± 0.57	2.69 ± 0.47	2.59 ± 0.57	3.871	0.024

Regarding the severity of infection risk after contact with patients having definite or suspected transmission routes, higher scores indicated greater awareness and concern regarding this risk. In the assessment of infection risk severity when contacting patients with non-communicable diseases, surgical departments scored significantly higher than internal medicine and other departments, suggesting a heightened level of vigilance. For situations involving contact with patients with intact skin and no invasive procedures, surgical departments again demonstrated higher scores, reflecting increased sensitivity to potential risk perception. When evaluating the severity of infection risk during medical activities involving patients with infectious diseases, surgical and other departments scored higher than internal medicine, indicating greater attentiveness to such risks. Finally, for procedures requiring maximum sterile barriers (e.g., catheter placement, puncture, surgery), surgical personnel assigned higher scores compared to internal medicine and other departments, highlighting their stronger emphasis on maintaining aseptic techniques.

As shown in Table 3, two factors were extracted through principal component analysis to assess the severity and significance of infection risks in clinical practice. This factor extraction was supported by scree plots and the interpret ability of the underlying constructs. The variance contribution rates of these two factors were 44.055 and 29.767%, respectively, demonstrating their substantial role in explaining the variability within the data-set. Furthermore, the significance factor exhibited a cumulative variance contribution rate of 51.505%, underscoring its considerable influence on the perception of infection risk significance in medical activities. For severity factor 1, the loading value for non-intravascular punctures, injections, and similar procedures was 0.876, indicating a high level of perceived severity regarding infection risks associated with these activities. Sterile liquid preparation had a loading of 0.866, highlighting the critical importance of aseptic techniques. Additionally, medical personnel with hand skin damage received a score of 0.748, emphasizing the relevance of personal hygiene practices in infection risk management.

**Table 3:** Factors were extracted according to severity and importance.

**Severity**	**Factor 1**	**Factor 2**	**Importance**	**Factor 1**
Severity of infection risk after various types of non-intravascular puncture, injection and other procedures and treatments	0.876	0.235	The importance of areas of medical activity where no patient resides or where the patient stays only briefly	0.766
Severity of infection risk after the occurrence of sterile liquid configuration	0.866	0.146	The importance of medical activities for medical staff with broken hand skin	0.748
Severity of infection risk after occurrence of medical staff with infectious diseases performing medical activities	0.835	0.176	The importance of contact with intact mucosa without entering sterile tissues, organs and blood flow of the human body, and without touching items with damaged skin and damaged mucosa	0.746
Severity of infection after exposure to a patient with a clear or suspected route of transmission	0.786	0.154	The importance of contact with patients with non-communicable infectious diseases	0.704
Severity of infection risk after occurrence of medical activities by medical staff with broken hand skin	0.763	0.349	Importance of performing catheter placement, puncture, surgery and other procedures that require maximum sterile barrier	0.614
Severity of infection risk after occurrence in non-sterile liquid configurations	0.755	0.385		
Severity of infection risk after exposure to intact mucosa without entering sterile human tissues, organs, and bloodstream, and without contact with damaged skin or damaged mucosa	0.692	0.469		
Severity of infection risk after use of medical fabrics with potential biological contamination risk by patients	0.651	0.512		
Severity of infection risk after exposure to non-infectious diseases	0.150	0.924		
Severity of infection risk after exposure to patients with intact skin and without performing any invasive procedures	0.198	0.909		
Severity of infection risk after exposure to no communicable infectious diseases	0.300	0.826		
Severity of infection risk in an area where the patient does not live or where the patient only stays for a short period of time	0.496	0.570		

The factor-loading scores with absolute values of ≥0.50 are bolded.

Factor 2 scores reflect the relative importance of various medical activity contexts in infection risk management. For instance, settings without patient residence or involving brief patient contact received a score of 0.766, underscoring the necessity for vigilance even in low-exposure environments. The severity scores for infection risks following exposure to patients with non-communicable diseases and contact with intact skin without invasive procedures were 0.150 and 0.198, respectively, indicating comparatively lower levels of perceived risk in these scenarios.

Among significance factors, contact with patients having a definite or suspected transmission route yielded a score of 0.786, highlighting the criticality of identifying and managing such routes to prevent infection spread. Similarly, procedures requiring maximum sterile barriers (e.g., catheter placement, puncture, surgery) scored 0.614, reinforcing the essential role of rigorous aseptic techniques during high-risk clinical interventions.

### 3.3 Linear regression analysis

Tables 4.1–4.5 employ linear regression analysis with the scores of each factor as the dependent variables and participants' general demographic information as the independent variables, aiming to explore the association between participant characteristics and key issues in the basic infection control risk assessment.

**Table 4.1:** Severity factor score Y (continuous variable).

**Factor**	**β**	**95% CI**	***P*-value**
Age	0.012	(−0.015, 0.039)	0.393
Gender = female	0.128	(−0.223, 0.479)	0.475
Occupation = nurse + medical technician	0.198	(−0.100, 0.496)	0.195
Department = surgical	0.625	(0.327, 0.923)	0.000
Department = others	0.428	(0.081, 0.775)	0.017
Professional title = intermediate	−0.001	(−0.334, 0.332)	0.996
Professional title = senior	−0.174	(−0.678, 0.330)	0.499

**Table 4.2:** Severity factor score Y1 (continuous variable).

**Factor**	**β**	**95% CI**	***P*-value**
Age	0.013	(−0.026, 0.052)	0.506
Gender = female	0.139	(−0.365, 0.643)	0.588
Occupation = nurse + medical technician	0.382	(−0.045, 0.809)	0.083
Department = surgical	0.576	(0.149, 1.003)	0.009
Department = others	0.423	(−0.075, 0.921)	0.098
Professional title = intermediate	0.151	(−0.327, 0.629)	0.538
Professional title = senior	0.031	(−0.694, 0.756)	0.934

**Table 4.3:** Severity factor score Y2 (continuous variable).

**Factor**	**β**	**95% CI**	***P*-value**
Age	0.01	(−0.029, 0.049)	0.623
Gender = female	0.111	(−0.393, 0.615)	0.666
Occupation = nurse + medical technician	−0.073	(−0.500, 0.345)	0.738
Department = surgical	0.698	(0.271, 1.125)	0.002
Department = others	0.434	(−0.064, 0.932)	0.091
Professional title = intermediate	−0.226	(−0.706, 0.245)	0.359
Professional title = senior	−0.478	(−1.203, 0.247)	0.199

**Table 4.4:** Importance factor score YZ (continuous variable).

**Factor**	**β**	**95% CI**	***P*-value**
Age	0.012	(−0.027, 0.051)	0.545
Gender = female	0.301	(−0.15, 0.752)	0.193
Occupation = nurse + medical technician	0.136	(−0.28, 0.552)	0.523
Department = surgical	0.919	(0.511, 1.327)	0.000
Department = others	0.295	(−0.152, 0.742)	0.199
Professional title = intermediate	−0.196	(−0.653, 0.261)	0.404
Professional title = senior	−0.446	(−1.163, 0.271)	0.227

**Table 4.5:** The possibility of infection during cannulation and maintenance of non-intravascular catheters into sterile tissues or organs (continuous variable).

**Factor**	**β**	**95% CI**	***P*-value**
Age	−0.011	(−0.058, 0.036)	0.659
Gender = female	0.298	(−0.27, 0.866)	0.307
Occupation = nurse + medical technician	0.272	(−0.249, 0.793)	0.308
Department = surgical	0.582	(0.063, 1.101)	0.03
Department = others	−0.286	(−0.868, 0.296)	0.338
Professional title = intermediate	−0.191	(−0.771, 0.389)	0.521
Professional title = senior	−0.053	(−0.917, 0.811)	0.904

As shown in Table 4.1, participants from surgical departments and other clinical departments exhibited statistically significant positive associations with the severity factor score. In contrast, variables such as age, sex, occupation (nursing or medical technical staff), and professional title did not demonstrate significant effects on the severity factor score. Furthermore, Tables 4.2–4.5 consistently indicate that participants from surgical departments had significant positive impacts on the severity factor scores Y1 and Y2 (both continuous variables), the importance factor score YZ (continuous variable), and the likelihood of infection risk associated with various types of non-endovascular catheter placement and maintenance into sterile tissues or organs (continuous variable).

### 3.4 Comprehensive analysis of reliability and validity

As presented in Table 5, the questionnaire was structured into four dimensions based on the content of the questions: importance, possibility, severity, and coping ability. The Cronbach's α coefficients obtained during the reliability analysis were 0.942, 0.976, 0.977, and 0.992, respectively, indicating a high level of internal consistency across all dimensions. To assess the questionnaire's validity, the Kaiser-Meyer-Olkin (KMO) measure and Bartlett's test of sphericity were employed. The KMO values for each dimension exceeded the threshold of 0.6, and the *P*-value from Bartlett's test of sphericity was below 0.001, which collectively suggest adequate construct validity. These results confirm that the questionnaire satisfies both reliability and validity requirements.

**Table 5:** Comprehensive analysis of reliability and validity.

**Objective**	**Number**	**Reliability**	**Validity**	
		**Cronbach's** α	**KMO**	**Bartlett test**
Significance	40	0.942	0.797	*P* < 0.001
Possibility	40	0.976	0.802	*P* < 0.001
Severity	40	0.977	0.707	*P* < 0.001
Resilience capacity	40	0.992	0.832	*P* < 0.001

## 4 Discussion

The study revealed that pediatric HCWs exhibited a relatively strong understanding of theoretical knowledge related to infection control. However, the mean score for peer knowledge was significantly lower, indicating that while individual knowledge may be sufficient, there is a deficiency in awareness of the collective understanding among colleagues. This gap in perception may impede effective communication and collaboration in the implementation of infection control practices, a finding consistent with those of previous studies (21).

Despite the existing knowledge and awareness of infection control measures, this study identified persistent challenges in the practical implementation of such practices. The findings revealed that demographic factors, including age, gender, and professional title, did not demonstrate a statistically significant correlation with infection control knowledge, indicating that these variables may not substantially influence attitudes toward infection control practices (22). In contrast, receiving NI-related education was identified as the most significant influencing factor on attitudes. Although certain factors such as age and gender are immutable, ongoing education regarding NIs remains crucial for enhancing not only individual knowledge but also for promoting a culture of shared learning and collaboration. Consistent with previous findings, this study demonstrated that participants who had received training within the past 5 years achieved higher knowledge scores (21). Enhancing educational knowledge through training healthcare workers and organizing workshops or seminars on the prevention, transmission, and control of NIs, as well as the implementation of infection control policies and practices, can contribute to reducing the spread of such infections in hospital settings (22). This approach has the potential to address gaps in peer knowledge and enhance the overall effectiveness of infection control practices (13–25).

The study revealed significant differences in infection control knowledge across various departments, with a notable disparity observed between surgical and internal medicine units. Healthcare personnel in surgical departments demonstrated a higher level of awareness regarding infection risks, as indicated by their consistently elevated severity scores across multiple scenarios. These findings are consistent with prior research suggesting that surgical settings inherently involve greater risks for NIs due to the nature of the procedures conducted (26). The observed variation in attitudes highlights the importance of implementing targeted educational interventions that are specifically tailored to the needs of individual departments. For example, healthcare personnel in internal medicine may benefit from intensified training programs focused on the distinct infection risks associated with their patient populations. By addressing the unique requirements and concerns of each department, healthcare institutions can promote a more proactive and effective approach to infection control.

The statistical analysis performed in this study, which included one-way ANOVA and linear regression, yielded meaningful insights into the factors influencing knowledge, attitudes, and practices (KAP) among pediatric healthcare workers (HCWs). The identification of 17 sub-questions with statistically significant differences underscores the variability in perceptions of severity and importance across different specialties. These results highlight the necessity for customized interventions that address the specific concerns and knowledge gaps prevalent within each department. Indeed, a study conducted in Italy reported higher compliance with infection control guidelines among nurses (15). Furthermore, the factor analysis identified two primary components associated with perceptions of severity and one component related to importance, suggesting that certain aspects of infection control are regarded as more critical than others. A clear understanding of these perceptions can inform the development of targeted educational materials and interventions that align with HCWs' professional experiences and concerns (27, 28).

This study is subject to several limitations. First, all variables were derived from self-reported data, which may introduce social-desirability and recall biases, potentially resulting in overestimation of favorable responses or obscuring areas of low compliance. Second, although the questionnaire demonstrated strong psychometric properties, it was not pilot-tested with front-line pediatric healthcare workers, which may have led to ambiguities in item phrasing or interpretation, thereby compromising response validity. Third, the investigation was conducted at a single tertiary children's hospital in Nanjing; consequently, findings may not be generalizable due to variations in institutional culture, staffing structures, patient demographics, regional epidemiology, and infection control practices across different settings. Multi-center studies encompassing diverse contexts are necessary to enhance external validity. Finally, while linear regression models were employed to analyze associations between demographic characteristics and factor scores, the manuscript does not provide documentation of diagnostic checks for key model assumptions—specifically linearity, homoscedasticity, normality of residuals, and absence of multicollinearity. Violations of these assumptions could bias coefficient estimates, standard errors, and confidence intervals. Future research should incorporate formal assumption testing or adopt robust or non-parametric analytical approaches as appropriate. Also, the questionnaire was not pilot-tested with the intended respondents. Although the items were refined through a Delphi panel and exhibited satisfactory internal consistency (as indicated by Cronbach's α) and adequate sampling suitability (as reflected in the KMO statistic), the absence of field piloting constrains our confidence in the instrument's comprehensibility, feasibility, and contextual relevance for the target population.

However, the study findings underscore the importance of ongoing research in the field of infection control, particularly within pediatric healthcare settings. Future studies should focus on developing and implementing targeted educational interventions aimed at improving KAP among HCWs. Additionally, longitudinal studies could provide insights into the long-term effectiveness of these interventions and their impact on infection rates. Moreover, exploring the barriers to effective implementation of infection control measures is crucial. Qualitative research methods, such as interviews and focus groups, can provide deeper insights into the challenges faced by HCWs and inform the development of strategies to overcome these obstacles.

## 5 Conclusion

This study offers meaningful insights into the KAP of pediatric HCWs concerning NI control in Nanjing, China. The statistically significant differences identified across specialties highlight the need for tailored educational interventions. By addressing the distinct needs and concerns of various departments, healthcare institutions can strengthen infection control practices, thereby contributing to improved patient safety and quality of care. Continued research and sustained educational efforts are crucial to cultivating a robust culture of infection prevention and control within pediatric healthcare environments.

## Data Availability

The raw data supporting the conclusions of this article will be made available by the authors, without undue reservation.
